# Classifying patients with lumbar spinal stenosis using painDETECT: a cross-sectional study

**DOI:** 10.1186/s12875-016-0486-z

**Published:** 2016-07-22

**Authors:** Naoto Takahashi, Osamu Shirado, Kazuki Kobayashi, Ryosuke Mashiko, Shin-ichi Konno

**Affiliations:** Department of Orthopaedic Surgery, Fukushima Medical University School of Medicine, Fukushima, Japan; Department of Pain Medicine, Fukushima Medical University School of Medicine, Fukushima, Japan; Department of Orthopaedic Surgery, Hoshi General Hospital, Koriyama, Japan; Department of Orthopaedic and Spinal Surgery, Aizu Medical Center Fukushima Medical University, Aizuwakamatsu, Japan; Department of Orthopaedic Surgery, Fukushima Prefectural Minami-Aizu Hospital, Minami Aizu, Japan

**Keywords:** Lumbar spinal stenosis, Nociceptive pain, Neuropathic pain, Cross-sectional study, painDETECT questionnaire, Numerical rating scale, Roland–Morris Disability Questionnaire score, Japanese Orthopaedic Association Back Pain Evaluation Questionnaire, 36-Item Short-Form Health Survey, Primary care

## Abstract

**Background:**

The pathological mechanisms of lumbar spinal stenosis are unclear. Family doctors in the primary care setting may perform medical examinations of patients with lumbar spinal stenosis. Our aim was to use the painDETECT questionnaire to quantify the pathological mechanisms of low back pain and/or leg pain caused by lumbar spinal stenosis.

**Methods:**

We enrolled 102 patients (37 men, 65 women) who had been newly diagnosed with lumbar spinal stenosis at 2 facilities. The patients’ conditions were evaluated using the painDETECT questionnaire, Numerical Rating Scale, Roland-Morris Disability Questionnaire, Japanese Orthopaedic Association Back Pain Evaluation Questionnaire, and 36-Item Short-Form Health Survey. The pathological mechanisms of low back pain and/or leg pain caused by lumbar spinal stenosis were classified based on results of the painDETECT questionnaire as nociceptive pain, neuropathic pain, or unclear type of pain (mixed pain). Statistical analyses were performed using the Kruskal–Wallis test. A value of *p* < 0.05 was considered to indicate statistical significance.

**Results:**

The mean age of all patients in this study was 70.3 ± 2 years. The male:female distribution was 37:65 (36.3:63.7 %). In all, 72 (70.6 %) patients had chronic pain (duration of ≥3 months), and 30 (29.4 %) had subacute or acute pain (duration of <3 months). The pain was classified as nociceptive in 59 patients (57.9 %), neuropathic in 18 (17.6 %), and unclear in 25 (24.5 %). The neuropathic pain group had a significantly lower quality of life (*p* < 0.05) than did the other groups.

**Conclusions:**

Patients with neuropathic back and/or leg pain caused by lumbar spinal stenosis may have lower physical and/or psychological quality of life than patients with such pain caused by other mechanisms.

## Background

Lumbar spinal stenosis (LSS) is the one of the most commonly encountered orthopedic disorders. Family doctors in the primary care setting may perform medical examinations of patients with LSS. Therefore, an understanding of the pathological mechanisms of LSS may be very helpful for these doctors.

LSS is defined as a reduction in the area of the spinal canal, lateral canal, and/or foramina. Symptoms of LSS may occur as a result of neurovascular mechanisms [1–3], such as reduced arterial flow in the cauda equina, venous congestion, increased epidural pressure, nerve root infiltration, and direct compression in the central canal and/or lateral recess [4]. Although the characteristic symptom of LSS is neurogenic intermittent claudication (NIC) [5, 6], other symptoms may appear as well, including low back pain (LBP), radicular pain and/or numbness down the leg, and motor weakness in the legs [7–10]. It is therefore important to analyze the pathological mechanisms of LBP and/or leg pain caused by LSS to alleviate the symptoms.

We considered the pain mechanisms according to a classification that included neuropathic pain, nociceptive pain, and mixed pain [11, 12]. Neuropathic pain is defined by the International Association for the Study of Pain as “pain initiated or caused by a primary lesion or dysfunction of the nervous system” [13]. It may be associated with abnormal sensations called dysesthesia or produced by normally nonpainful stimuli such as allodynia. Nociceptive pain may also be a result of the encoding and processing of harmful stimuli in the nervous system, reflecting the ability of the body to sense potential harm [14]. Because the developmental mechanisms responsible for neuropathic and nociceptive pain differ, treatment plans differ as well. Therefore, the pathological mechanisms of the pain should be precisely identified to arrive at an exact diagnosis of LSS-derived LBP and/or leg pain.

A recent study using the painDETECT questionnaire demonstrated that neuropathic pain was less common in patients with chronic LBP and that patients with neuropathic back and/or leg pain reported significantly more pain, disability, anxiety, depression, and reduced quality of life (QOL) than patients with nociceptive pain [15]. The pathological mechanisms of LSS, however, were unclear. Few studies have used the painDETECT questionnaire to quantify the pathological mechanisms of LSS [16, 17]. The purpose of this study was to quantify the pathological mechanisms of LBP and/or leg pain caused by LSS using the painDETECT questionnaire, and to report quality of life data in patients with LSS. This study may help to fully elucidate the epidemiology of LSS-derived LBP and/or leg pain in the primary care setting.

## Methods

The ethics committees of the participating research institutions approved this study. Written informed consent was obtained from all patients prior to their participation.

This cross-sectional multicenter observational study was conducted nationwide at two hospitals with their attending spinal surgeons. The recruitment period was 1.5 years (1 April 2013 to 30 September 2014).

### Patients

The study group included 102 consecutive patients (37 men, 65 women) who were newly diagnosed with LSS at two facilities: Aizu Medical Center Fukushima Medical University and the Fukushima Prefectural Minami-Aizu Hospital. Three spinal surgeons diagnosed the patients with LSS based on magnetic resonance imaging (MRI) findings of LSS, subjective symptoms, and neurological findings. All of the patients had subjective symptoms that included leg symptoms, LSS-related neurological findings, and MRI evidence of LSS. An independent radiologist assessed the MRI scans obtained for each patient at the time of diagnosis for evidence of LSS, including central, lateral recess, and foraminal stenosis. The MRI diagnosis of LSS was based on the following findings: (1) blockage of cerebrospinal fluid and/or the disappearance of fatty tissue surrounding the foramen on T1-weighted images in the sagittal view; (2) central stenosis, lateral recess stenosis, and/or foraminal stenosis caused by lumbar disc compression from the front, and/or increased size of the ligamentum flavum, and/or articular factors from the back in the axial view; and/or (3) compression of the nerve root surrounding the foramen in the coronal view. The ankle-brachial pressure index (ABI) was also checked in all patients to distinguish NIC from vascular intermittent claudication (ABI <0.9). The ABI is the systolic pressure at the ankle divided by the systolic pressure at the arm. It has been shown to be a specific and sensitive metric for the diagnosis of peripheral arterial disease [18].

The inclusion criteria for all patients were (1) a diagnosis of lumbar spondylosis, degenerative spondylolisthesis, or degenerative scoliosis with LSS; (2) pain and/or numbness in the lumbar dermatomal distribution; (3) motor or sensory neurological signs (hypoesthesia, hyperesthesia, allodynia, or dysesthesia) in the affected dermatomes; (4) cognitive capability to satisfy our inquiries; and (5) neurogenic NIC due to LSS. The exclusion criteria for all patients were (1) diagnosis of lumbar degenerative disease without LSS; (2) predominantly axial spinal pain; (3) rheumatoid arthritis; (4) known peripheral neuropathy; (5) history of workmen’s compensation or disability issues; (6) chronic depression and use of antidepressant medication; and (7) patients with an ABI of <0.9.

### Methods

The painDETECT neuropathic pain screening questionnaire scores [17] were used to identify the three pain subgroups of patients with LBP and/or leg pain caused by LSS: (1) those with nociceptive pain, (2) those with neuropathic pain, and (3) those in whom the type of pain was unclear [15, 19]. We used the Japanese version of the painDETECT neuropathic pain screening questionnaire score [20]. The cut-off values for categorizing the type of pain based on the painDETECT neuropathic pain screening questionnaire scores were as follows: nociceptive pain, 0–12; unclear type of pain (mixed nociceptive and neuropathic pain), 13–18; and neuropathic pain, 19–38. The painDETECT neuropathic pain screening questionnaire is a reliable screening tool with high sensitivity, high specificity, and positive predictive accuracy. These parameters were each 84 % in a palm-top computerized version of the questionnaire and 85, 80, and 83 %, respectively, in a corresponding pencil-and-paper questionnaire [17]. Matsubayashi et al. [20] demonstrated that the Japanese version of the painDETECT neuropathic pain screening questionnaire has good validity and reliability, similar to that found with the original version.

Numerical rating scale (NRS) and Roland-Morris Disability Questionnaire (RDQ) scores [21] were used to compare the severity of subjective symptoms and the QOL associated with LBP at the patient’s first medical examination. The patients used the NRS for self-evaluation of their leg pain and/or numbness. Both the NRS and RDQ scores were evaluated at the time of the first medical examination. We used the Japanese version of the RDQ score [22].

The Japanese Orthopaedic Association Back Pain Evaluation Questionnaire (JOABPEQ) [23, 24] and the 36-Item Short-Form Health Survey (SF-36) [25] were used to compare the severity of subjective symptoms and the QOL associated with LBP and health at the patient’s first medical examination. The JOABPEQ has five subscales, and the SF-36 has eight. For both questionnaires, higher scores indicate better QOL. We used the Japanese version of the SF-36 [26, 27]. The SF-36 uses scores of 0 to 100, but the scores are not based on the norm.

The primary outcome was the type of pain—nociceptive, neuropathic, or unclear (mixed)—in patients with LBP and/or leg pain caused by LSS. At the first medical examination, we compared the patients’ demographic and clinical characteristics; NRS (LBP); NRS (leg pain, leg numbness); and RDQ, JOABPEQ, and SF-36 scores among the three pain groups using the Kruskal–Wallis test. We also compared these outcomes according to the duration of symptoms—i.e., <3 months (acute and/or subacute phase) or ≥3 months (chronic phase) after the onset of LBP and/or leg pain caused by LSS. A *p* value of <0.05 was considered to indicate statistical significance. The statistical analyses were performed using StatView 5.0 statistical software (SAS Institute, Cary, NC, USA). The statistical power analysis of this study was performed using G*Power 3.1 (Heinrich-Heine-Universität Düsseldorf, Düsseldorf, Germany) [28]. The power analysis utilized an effect size of 0.4, alpha level of 0.05, power (1 − beta error probability) of 0.95, and requirement of three groups. Finally, the total required sample size was calculated as 102.

### Ethics

This study was approved by the ethics committees of the two participating research institutions: Aizu Medical Center Fukushima Medical University and Fukushima Prefectural Minami-Aizu Hospital. Informed consent was obtained from all patients.

## Results

The mean age of all patients (*n* = 102) in this study was 70.3 ± 2 years. The male:female distribution was 37:65 (36.3:63.7 %). The NIC was radicular in 68.6 % (*n* = 70), caudal in 8.8 % (*n* = 9), and mixed in 22.6 % (*n* = 23). Spinal stenosis seen by MRI appeared at one level in 41.4 % (*n* = 42), two levels in 43.1 % (*n* = 44), and three levels in 11.7 % (*n* = 12). The causes of LSS were lumbar spondylitis in 55.0 % (*n* = 56), degenerative spondylolisthesis in 33.3 % (*n* = 34), and degenerative scoliosis in 11.7 % (*n* = 12).

The demographic and clinical characteristics of the patients with LBP and/or leg pain caused by LSS for each pain subgroup are shown in Table 1. The demographic and clinical characteristics of the patients with LBP and/or leg pain caused by LSS were similar among the three pain subgroups.

**Table 1:** Demographic and clinical characteristics at the first medical examination

Characteristic	Nociceptive pain	Type of pain unclear (mixed pain)	Neuropathic pain	*p* ^a^
Age (years)	71.4 ± 1.36	69.0 ± 2.34	68.3 ± 2.91	0.720
Sex				0.572
Male	18	11	8	
Female	41	14	10	
Duration of pain				0.914
≧3 months	41	17	14	
<3 months	18	8	4	
Affected spinal level				0.730
L3–L4	18	9	3	
L4–L5	36	16	15	
L5–S1	5	0	0	
NIC type				0.668
Radicular	41	15	14	
Caudal	5	4	0	
Mixed	13	6	4	
Spinal stenosis levels involved^b^				0.373
1	28	8	6	
2	25	11	8	
≥3	6	6	4	
Cause of LSS				0.672
Lumbar spondylitis	30	15	11	
Degenerative spondylolisthesis	20	8	6	
Degenerative scoliosis	9	2	1	

The demographic and clinical characteristics of the patients with LBP and/or leg pain caused by LSS were similar among the three pain subgroups

*LBP* low back pain, *LSS* lumbar spinal stenosis, *NIC* neurogenic intermittent claudication, *LDH* lumbar disc degeneration, *MRI* magnetic resonance imaging

^a^Kruskal–Wallis test

^b^Seen on MRI

Among all patients with LBP and/or leg pain caused by LSS, 59 (57.9 %) had nociceptive pain, 18 (17.6 %) had neuropathic pain, and 25 (24.5 %) had an unclear type of pain (mixed pain) at their first medical examination (Table 2). In all, 72 (70.6 %) patients had chronic pain (duration of ≥3 months), and 30 (29.4 %) had subacute or acute pain (duration of <3 months). Among the patients whose LBP and/or leg pain caused by LSS had been present for ≥3 months, 41 (56.9 %) had nociceptive pain, 14 (19.5 %) had neuropathic pain, and 17 (23.6 %) had pain of unclear (mixed pain) at their first medical examination (Table 2). Among those whose LBP and/or leg pain caused by LSS had been present for <3 months, 18 (60.0 %) patients had nociceptive pain, 4 (13.3 %) had neuropathic pain, and 8 (26.7 %) had unclear pain (mixed pain) at their first medical examination (Table 2). The populations of the three pain subgroups [nociceptive, neuropathic, and unclear (mixed)] with LBP and/or leg symptoms caused by LSS (regardless of pain duration) were thus similar.

**Table 2:** painDETECT questionnaire scores at the first medical examination

Origin of pain	Patients, n (%)
Total patients	102 (100)
Nociceptive pain	59 (57.9)
Type of pain unclear (mixed pain)	25 (24.5)
Neuropathic pain	18 (17.6)
Group with pain present ≥3 months	
Nociceptive pain	41 (56.9)
Type of pain unclear (mixed pain)	17 (23.6)
Neuropathic pain	14 (19.5)
Group with pain present <3 months	
Nociceptive pain	18 (60.0)
Type of pain unclear (mixed pain)	8 (26.7)
Neuropathic pain	4 (13.3)

*LBP* low back pain, *LSS* lumbar spinal stenosis

NRS scores for LBP, leg pain, and leg numbness in patients with LBP and/or leg pain caused by LSS were not significantly different among the three pain groups (Table 3). However, the RDQ score in patients with LBP and/or leg pain caused by LSS was significantly lower in the neuropathic pain group than in the other groups (*p* < 0.05) (Table 3). In contrast, there were no significant differences among the three pain groups regarding (1) the NRS scores for LBP, leg pain, or leg numbness or (2) the RDQ scores in patients with LBP and/or leg pain caused by LSS that had been present for ≥3 months or <3 months (Table 3).

**Table 3:** NRS and RDQ scores at the first medical examination

Parameter	Nociceptive pain	Type of pain unclear (mixed pain)	Neuropathic pain	*p* ^a^
Total patients with pain at first examination				
NRS (low back pain)	4.75 ± 0.433	5.88 ± 0.561	6.39 ± 0.691	0.099
NRS (leg pain)	5.59 ± 0.397	6.72 ± 0.618	6.39 ± 0.687	0.205
NRS (leg numbness)	4.88 ± 0.422	5.56 ± 0.651	6.83 ± 0.556	0.101
RDQ	9.00 ± 0.760	9.52 ± 1.16	6.68 ± 1.57	0.025^a^
Group with pain present ≥3 months				
NRS (low back pain)	5.27 ± 0.523	6.12 ± 0.587	6.43 ± 0.724	0.462
NRS (leg pain)	5.61 ± 0.479	6.35 ± 0.722	6.57 ± 0.685	0.566
NRS (leg numbness)	5.49 ± 0.513	5.82 ± 0.666	6.50 ± 0.618	0.713
RDQ	9.66 ± 0.952	8.82 ± 1.41	13.7 ± 1.77	0.074
Group with pain present <3 months				
NRS (low back pain)	3.56 ± 0.715	5.38 ± 1.28	6.25 ± 2.06	0.275
NRS (leg pain)	5.56 ± 0.729	7.50 ± 1.20	5.75 ± 2.18	0.275
NRS (leg numbness)	3.50 ± 0.643	5.00 ± 1.52	8.00 ± 1.23	0.060
RDQ	7.50 ± 1.19	11.0 ± 2.09	13.5 ± 3.97	0.125

Data are shown as mean ± standard error

The NRS scores of LBP, leg pain, and leg numbness in patients with LBP and/or leg pain caused by LSS were not significantly different among the three pain groups. However, the RDQ score in patients with LBP and/or leg pain caused by LSS was significantly lower in the neuropathic pain group than in the other groups (*p* < 0.05)

*NRS* numerical rating scale, *RDQ* Roland–Morris Disability Questionnaire

^a^Kruskal–Wallis test

The five JOABPEQ subscales were LBP, lumbar function, walking ability, social life function, and mental health. The eight SF-36 subscales were physical functioning, physical role functioning, bodily pain, general health perceptions, vitality, emotional functioning, social role functioning, and mental health. The subscale scores of both questionnaires for each pain group are shown in Tables 4 and 5. There were statistically significant differences in three JOABPEQ subscales [lumbar function, social life function, and mental health (*p* < 0.05)] and in two SF-36 subscales [physical function and bodily pain (*p* < 0.05)] (Tables 4 and 5). In regard to pain duration, for patients whose pain had been present for ≥3 months after the onset of symptoms, there were statistically significant differences in two JOABPEQ subscales [lumbar function and mental health (*p* < 0.05)], but no statistically significant differences in any of the SF-36 subscales (Tables 4 and 5). For patients whose pain had been present for <3 months after the onset of symptoms, there were no statistically significant differences in any of the JOABPEQ subscales (*p* < 0.05), but there was a statistically significant difference in one SF-36 subscale [bodily pain (*p* < 0.05)] (Tables 4 and 5).

**Table 4:** JOABPEQ score for each pain type at the first medical examination

Parameter	Nociceptive pain	Type of pain unclear (mixed pain)	Neuropathic pain	*p* ^a^
Total patients with pain at first examination				
Low back pain	50.8 ± 4.21	41.1 ± 7.30	30.9 ± 6.94	0.071
Lumbar function	66.1 ± 3.66	64.9 ± 5.29	37.0 ± 7.80	0.005^a^
Walking ability	44.8 ± 4.06	40.0 ± 4.37	35.3 ± 6.66	0.580
Social life function	50.6 ± 2.99	36.8 ± 3.45	42.8 ± 5.79	0.015^a^
Mental health	49.4 ± 2.47	40.6 ± 3.54	39.8 ± 5.17	0.048^a^
Group with pain present ≥3 months				
Low back pain	48.1 ± 5.07	41.1 ± 8.23	29.6 ± 8.13	0.188
Lumbar function	63.6 ± 4.91	65.1 ± 6.87	38.1 ± 8.56	0.033^a^
Walking ability	42.6 ± 5.04	39.5 ± 6.00	36.7 ± 7.06	0.881
Social life function	49.5 ± 3.91	35.5 ± 4.30	44.1 ± 6.79	0.064
Mental health	52.5 ± 2.81	41.1 ± 4.42	40.7 ± 5.60	0.032^a^
Group with pain present <3 months				
Low back pain	57.2 ± 7.55	41.0 ± 15.6	35.8 ± 14.8	0.373
Lumbar function	71.8 ± 4.24	64.5 ± 8.42	33.3 ± 20.8	0.172
Walking ability	49.7 ± 6.80	41.3 ± 5.41	30.5 ± 19.1	0.530
Social life function	53.2 ± 4.17	39.5 ± 6.04	38.5 ± 12.2	0.204
Mental health	42.2 ± 4.62	39.8 ± 6.26	36.5 ± 14.2	0.869

Data are shown as mean ± standard error

The JOABPEQ comprises five subscales. Higher scores indicate better quality of life

There were statistically significant differences in three JOABPEQ subscale scores [lumbar function, social life function, and mental health (*p* < 0.05)]. With respect to pain duration, for patients with chronic pain, there were statistically significant differences in two JOABPEQ subscale scores [lumbar function and mental health (*p* < 0.05)]. For patients with subacute or acute pain, there were no statistically significant differences in any of the JOABPEQ subscale scores (*p* < 0.05)

*JOABPEQ* Japanese Orthopaedic Association Back Pain Evaluation Questionnaire

^a^Kruskal–Wallis test

**Table 5:** SF-36 score for each pain type at the first medical examination

Parameter	Nociceptive pain	Type of pain unclear (mixed pain)	Neuropathic pain	*p* ^a^
Total patients with pain at first examination				
Physical functioning	27.0 ± 2.14	30.2 ± 2.67	21.1 ± 4.73	0.040^a^
Role physical	33.8 ± 2.15	34.5 ± 3.05	26.5 ± 4.13	0.326
Bodily pain	35.8 ± 0.92	32.5 ± 1.64	29.6 ± 2.53	0.011^a^
General health	42.4 ± 1.20	41.4 ± 1.99	39.5 ± 2.87	0.668
Vitality	46.3 ± 1.43	41.6 ± 3.01	44.1 ± 2.73	0.396
Social functioning	44.3 ± 1.66	40.5 ± 2.55	37.0 ± 4.53	0.179
Role emotional	40.1 ± 2.16	36.5 ± 3.09	32.4 ± 4.04	0.160
Mental health	46.3 ± 1.40	42.8 ± 2.61	42.1 ± 3.39	0.287
Group with pain present ≥3 months				
Physical functioning	25.5 ± 2.58	28.4 ± 3.28	21.9 ± 5.29	0.201
Role physical	32.2 ± 2.86	33.0 ± 4.15	28.2 ± 4.61	0.797
Bodily pain	34.9 ± 1.11	30.9 ± 1.23	31.4 ± 2.96	0.103
General health	42.2 ± 1.42	38.1 ± 1.85	39.8 ± 3.12	0.318
Vitality	45.1 ± 1.72	38.9 ± 3.88	45.0 ± 3.18	0.471
Social functioning	44.1 ± 2.00	39.7 ± 3.24	39.2 ± 5.09	0.473
Role emotional	39.0 ± 2.88	35.1 ± 4.10	34.6 ± 4.49	0.522
Mental health	46.6 ± 1.79	40.0 ± 3.20	44.0 ± 3.64	0.183
Group with pain present <3 months				
Physical functioning	30.5 ± 3.80	34.0 ± 4.61	18.2 ± 11.9	0.197
Role physical	37.3 ± 2.60	37.9 ± 3.69	20.4 ± 9.89	0.166
Bodily pain	37.8 ± 1.58	35.9 ± 4.35	23.2 ± 3.55	0.021^a^
General health	42.7 ± 2.29	48.5 ± 3.87	38.2 ± 7.90	0.335
Vitality	49.2 ± 2.53	47.6 ± 4.12	41.0 ± 5.76	0.494
Social functioning	44.7 ± 3.10	42.3 ± 4.26	29.2 ± 10.2	0.288
Role emotional	42.7 ± 2.68	39.6 ± 4.33	24.7 ± 9.25	0.121
Mental health	45.6 ± 2.19	48.8 ± 3.98	35.2 ± 8.49	0.292

Data are shown as mean ± standard error

The SF-36 comprises eight subscales (score of 0–100). Higher scores indicate better quality of life

There were statistically significant differences in two SF-36 subscale scores [physical function and bodily pain (*p* < 0.05)]. With respect to pain duration, for patients with chronic pain, there were no statistically significant differences in any of the SF-36 subscale scores. For patients with subacute or acute pain, there was a statistically significant difference in one SF-36 subscale score [bodily pain (*p* < 0.05)]

*SF-36* 36-Item Short-Form Health Survey

^a^Kruskal–Wallis test

## Discussion

The present study demonstrated four major points. (1) Overall, 58 % of the 102 patients with LBP and/or leg pain caused by LSS had nociceptive pain, 18 % had neuropathic pain, and 24 % had an unclear type of pain (mixed pain) at their first medical examination. (2) NRS scores for LBP, leg pain, and leg numbness in patients with LBP and/or leg pain caused by LSS were not significantly different among the three pain groups. The RDQ score in patients with LBP and/or leg pain caused by LSS, however, was significantly lower in the neuropathic pain group than in the other groups. (3) Three JOABPEQ subscales (lumbar function, social life function, and mental health) were significantly lower in the neuropathic pain group than in the other groups. (4) Two SF-36 subscales (physical function and bodily pain) were significantly lower in the neuropathic pain group than in the other groups.

The statistical power analysis performed in this study indicated a required total sample size of 102, and the power was 0.95. Therefore, we believe that the power of this study was adequate.

LSS may occur at different levels in the spinal canal. It may be caused by entrapment of nerve roots in the cauda equina due to hypertrophy of the osseus and soft tissue structures surrounding the lumbar spinal canal. Central canal stenosis may compress nerve roots in the cauda equina, whereas lateral recess stenosis and/or foraminal stenosis may compress nerve roots but spare the spine [29, 30]. Although the lower limb symptoms associated with LSS are mainly attributed to mechanoreceptive compression of nerve rootlets and/or the cauda equina, they are also associated with inflammation, ischemia, malnutrition, nerve degeneration, and nerve injury. They consequently have a complicated pathophysiology. The pathological mechanisms of lower limb symptoms caused by LSS involve nociceptive, inflammatory, and/or neuropathic pain components, which may result from postural changes or persistent compression of the nerve roots and/or cauda equina while walking.

The prevalence of neuropathic pain in the general population is unclear, although it has been reported at 3–9 % based on the results of various screening questionnaires in Europe and the United States [31–34]. Few studies, however, have used the painDETECT to analyze the pathological mechanisms of LBP and/or leg pain caused by LSS in primary care-referred patients. Beith et al. [15] studied patients from southeastern England who had LBP with or without leg pain and had been referred for physiotherapy. The authors reported that 59 % of the patients reported what was identified to be nociceptive pain, 16 % had neuropathic pain, and 25 % had an unclear type (mixed pain). This result is very similar to that obtained in the present study of the pathological mechanisms of LSS-derived LBP and/or leg pain (nociceptive pain, 58 %; neuropathic pain, 18 %; unclear pain (mixed pain), 24 %). Therefore, our findings may accurately elucidate the pathological mechanisms of LSS-derived LBP and/or leg pain in the primary care setting.

The NRS scores for LBP, leg pain, and leg numbness in our patients with LBP and/or leg pain caused by LSS were not significantly different among the three pain groups. However, the RDQ score, three JOABPEQ subscale scores (lumbar function, social life function, and mental health), and two SF-36 subscale scores (physical function and bodily pain) were significantly lower in the neuropathic pain group than in the other groups. These data suggest that the neuropathic pain component produced significantly lower scores than its non-neuropathic pain components and reduced the patients’ physical and/or psychological QOL. Therefore, patients with neuropathic back and/or leg pain caused by LSS should be diagnosed as soon as possible after referral. A previous study [35] compared JOABPEQ scores between patients with LBP who experienced either neuropathic pain or nociceptive pain as assessed by the Japanese version of the painDETECT. Their findings suggest that neuropathic pain affects the social and psychological well-being of patients with LBP and demonstrate that patients with neuropathic back and/or leg pain caused by LSS might have particularly low physical and/or psychological QOL.

Finally it is considered that the impact of this study on education, health services and research regarding primary care would suggest that it may be very important to elucidate the pathological mechanisms and the epidemiology of LSS-derived LBP and/or leg pain by classifying the patients with LSS using painDETECT in order to analyze either neuropathic pain or non-neuropathic pain, and this may be helpful to examine how to treatment for LSS-derived LBP and/or leg pain in primary care setting.

The present study has some limitations that require attention. First, we studied only a small population, although we believe that the power was adequate in this study. Future studies must plan to evaluate a larger population. Second, this study had a cross-sectional design—it was not a longitudinal study. We did not evaluate therapeutic efficacy for LSS in this study. Hence, future studies should evaluate the therapeutic efficacy for each pain group caused by LSS, including conservative versus surgical therapy. Third, it was considered the lack thorough clinical and laboratory investigation in this study, since our study utilizes only questionnaires to classify the pain introduced by LSS. A clinical study with post-licensure surveillance should be implemented, ideally by setting up a database that includes all patients seeking treatment for LBP and/or leg pain caused by LSS, minimizing losses to follow-up, and using validated methods to gather clinically relevant data including demographic information, clinical features, common co-morbidities, conservative and/or surgical treatments applied to each patient, experience and training standards of the care providers applying each treatment, and each patient’s clinical evolution [36].

## Conclusions

It may be important to analyze the pathological mechanisms of neuropathic pain and non-neuropathic pain in patients with LBP and/or leg pain caused by LSS. Patients with neuropathic back and/or leg pain caused by LSS may experience particularly low physical and/or psychological QOL.

## Abbreviations

ABI, ankle-brachial pressure index; JOABPEQ, Japanese Orthopaedic Association Back Pain Evaluation Questionnaire; LBP, low back pain; LSS, lumbar spinal stenosis; MRI, magnetic resonance imaging; NIC, neurogenic intermittent claudication; NRS, numerical rating scale; QOL, quality of life; RDQ, Roland–Morris Disability Questionnaire; SF-36, 36-Item Short-Form Health Survey

## References

[1] Porter RW (1996). Spinal stenosis and neurogenic claudication. Spine.

[2] Takahashi K, Kagechika K, Takino T, Matsui T, Miyazaki T, Shima I (1995). Changes in epidural pressure during walking in patients with lumbar spinal stenosis. Spine.

[3] Takahashi K, Miyazaki T, Takino T, Matsui T, Tomita K (1995). Epidural pressure measurements: relationship between epidural pressure and posture in patients with lumbar spinal stenosis. Spine.

[4] Kobayashi S, Kokubo Y, Uchida K, Yayama T, Takeno K, Negoro K (2005). Effect of lumbar nerve root compression on primary sensory neurons and their central branches: changes in the nociceptive neuropeptides substance P and somatostatin. Spine.

[5] Verbiest H (1954). A radicular syndrome from developmental narrowing of the lumbar vertebral canal. J Bone Joint Surg (Br).

[6] Verbiest H (1955). Further experiences on the pathological influence of a developmental narrowness of the bony lumbar vertebral canal. J Bone Joint Surg (Br).

[7] Kikuchi S, Hasue M (1985). Clinical analyses of neurogenic intermittent claudication in lumbar spine diseases. Orthop Trans.

[8] Kikuchi S, Hoshika I, Matsui T, Hasue M (1986). [Neurogenic intermittent claudication in lumbar spine disease: Part 1.]. Orthop Surg.

[9] Kikuchi S, Hasue M (1988). Treatment of neurogenic intermittent claudication in degenerative stenosis. Orthop Trans.

[10] Sato S, Kikuchi S (1997). Clinical analysis of two-level compression of the cauda equina and the nerve roots in lumbar spinal canal stenosis. Spine.

[11] Audette JF, Emenike E, Meleger AL (2005). Neuropathic low back pain. Curr Pain Headache Rep.

[12] Jensen TS, Baron R (2003). Translation of symptoms and signs into mechanisms in neuropathic pain. Pain.

[13] Mersky H, Bogduk N, International Association for the Study of Pain (1994). Part 3. Pain terms, a current list with definitions and notes on usage. Classification of chronic pain. IASP Task Force on Taxonomy.

[14] Loeser JD, Trede RD (2008). The Kyoto protocol of IASP Basic Pain Terminology. Pain.

[15] Beith ID, Kemp A, Kenyon J, Prout M, Chestnut TJ (2011). Identifying neuropathic back and leg pain: a cross-sectional study. Pain.

[16] Rados I, Sakic Zdravcevic K, Hrgovic Z (2013). painDETECT questionnaire and lumbar epidural steroid injection for chronic radiculopathy. Euro Neurol.

[17] Freynhagen R, Baron R, Gockel U, Tölle TR (2006). PainDETECT: a new screening questionnaire to identify neuropathic components in patients with back pain. Curr Med Res Opin.

[18] Ai-Qaisi M, Nott DM, King DH, Kaddoura S (2009). Ankle brachial pressure index (ABPI): An update for practitioners. Vasc Health Risk Manag.

[19] Morsø L, Kent PM, Albert HB (2011). Are self-reported pain characteristics, classified using the painDETECT questionnaire, predictive of outcome in people with low back pain and associated leg pain?. Clin J Pain.

[20] Matsubayashi Y, Takeshita K, Sumitani M, Oshima Y, Tonosu J, Kato S (2013). Validity and reliability of the Japanese version of the painDETECT questionnaire: a multicenter observational study. PLoS One.

[21] Roland M, Fairbank J (2000). The roland-morris disability questionnaire and the oswestry disability questionnaire. Spine.

[22] Suzukamo Y, Fukuhara S, Kikuchi S, Konno S, Roland M, Iwamoto Y (2003). Validation of the japanese version of the roland-morris disability questionnaire. J Orthop Sci.

[23] Fukui M, Chiba K, Kawakami M, Kikuchi S, Konno S, Miyamoto M (2007). Japanese orthopaedic association back pain evaluation questionnaire. Part 2. Verification of its reliability: the subcommittee on low back pain and cervical myelopathy evaluation of the clinical outcome committee of the japanese orthopaedic association. J Orthop Sci.

[24] Fukui M, Chiba K, Kawakami M, Kikuchi S, Konno S, Miyamoto M (2008). Japanese orthopaedic association back pain evaluation questionnaire. Part 3. Validity study and establishment of the measurement scale: subcommittee on low back pain and cervical myelopathy evaluation of the clinical outcome committee of the Japanese Orthopaedic Association, Japan. J Orthop Sci.

[25] Larson JS, The MOS (1997). 36-Item Short Form Health Survey: a conceptual analysis. Eval Health Prof.

[26] Fukuhara S, Bito S, Green J, Hsiao A, Kurokawa K (1998). Translation, adaption, and validation of the SF-36 Health Survey for use in Japan. J Clin Epidemiol.

[27] Fukuhara S, Ware JE, Kosinski M, Eada S, Gandek B (1998). Psychometric and clinical tests of validity of the Japanese SF-36 Health Survey. J Clin Epidemiol.

[28] Faul F, Erdfelder E, Lang A-G, Buchner A (2007). G*Power 3: A flexible statistical power analysis program for the social, behavior, and biomedical sciences. Behav Res Methods.

[29] Alvarez JA, Hardy RH (1998). Lumbar spine stenosis: a common cause of back and leg pain. Am Fam Physician.

[30] Szpalski M, Gunzburg R (2003). Lumbar spinal stenosis in the elderly: an overview. Eur Spine J.

[31] Bouhassira D, Lantéri-Minet M, Attal N, Laurenr B, Touboul C (2008). Prevalence of chronic pain with neuropathic characteristics in the general population. Pain.

[32] Gustorff B, Dorner T, Likar R, Grisold W, Lawrence K, Schwarz F (2008). Prevalence of self-reported neuropathic pain and impact on quality of life: a prospective representative survey. Acta Anaesthesiol Scand.

[33] Torrance N, Smith B, Bennett M, Lee A (2006). The epidemiology of chronic pain of predominantly neuropathic origin: results from a general population survey. J Pain.

[34] Yawn B, Wollan P, Weibgarten T, Watson J, Hooten W, Melton L (2009). The prevalence of neuropathic pain: clinical evaluation compared with screening tools in a community population. Pain Med.

[35] Hiyama A, Watanabe M, Katoh H, Sato M, Sakai D, Mochida J (2015). Evaluation of quality of life and neuropathic pain in patients with low back pain using the Japanese Orthopedic Association Back Pain Evaluation Questionnaire. Eur Spine J.

[36] Carragee EJ, Deyo RA, Kovacs FM, Peul WC, Lurie JD, Urrútia G (2009). Clinical research: is the spine field a mine field?. Spine.

